# Supplementary material for: The transcriptional response of Yersinia pseudotuberculosis to macrophage-released metabolites during growth within synthetic microcolonies
Source: mBio. 2026 Sep 21;17(10):e00691-26. doi: 10.1128/mbio.00691-26 (PMC13712848; doi:10.1128/mbio.00691-26)
Supplement: Supplementary file 1 [file mbio.00691-26-s0005.pdf]

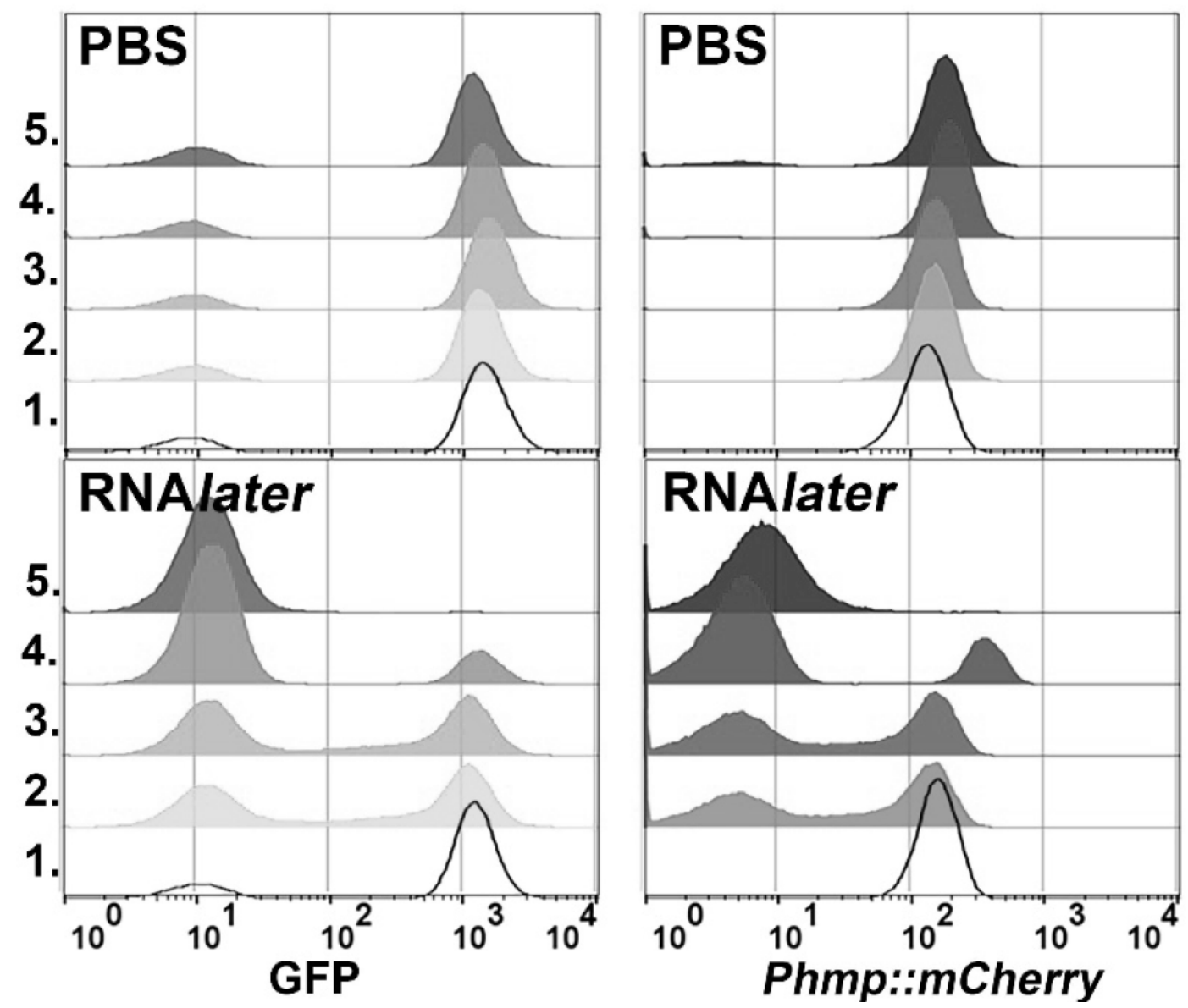
PBS
PBS
RNA*later*
RNA*later*
5.
4.
3.
2.
1.
5.
4.
3.
2.
1.
10^0 10^1 10^2 10^3 10^4
GFP
10^0 10^1 10^2 10^3 10^4
*Phmp::mCherry*

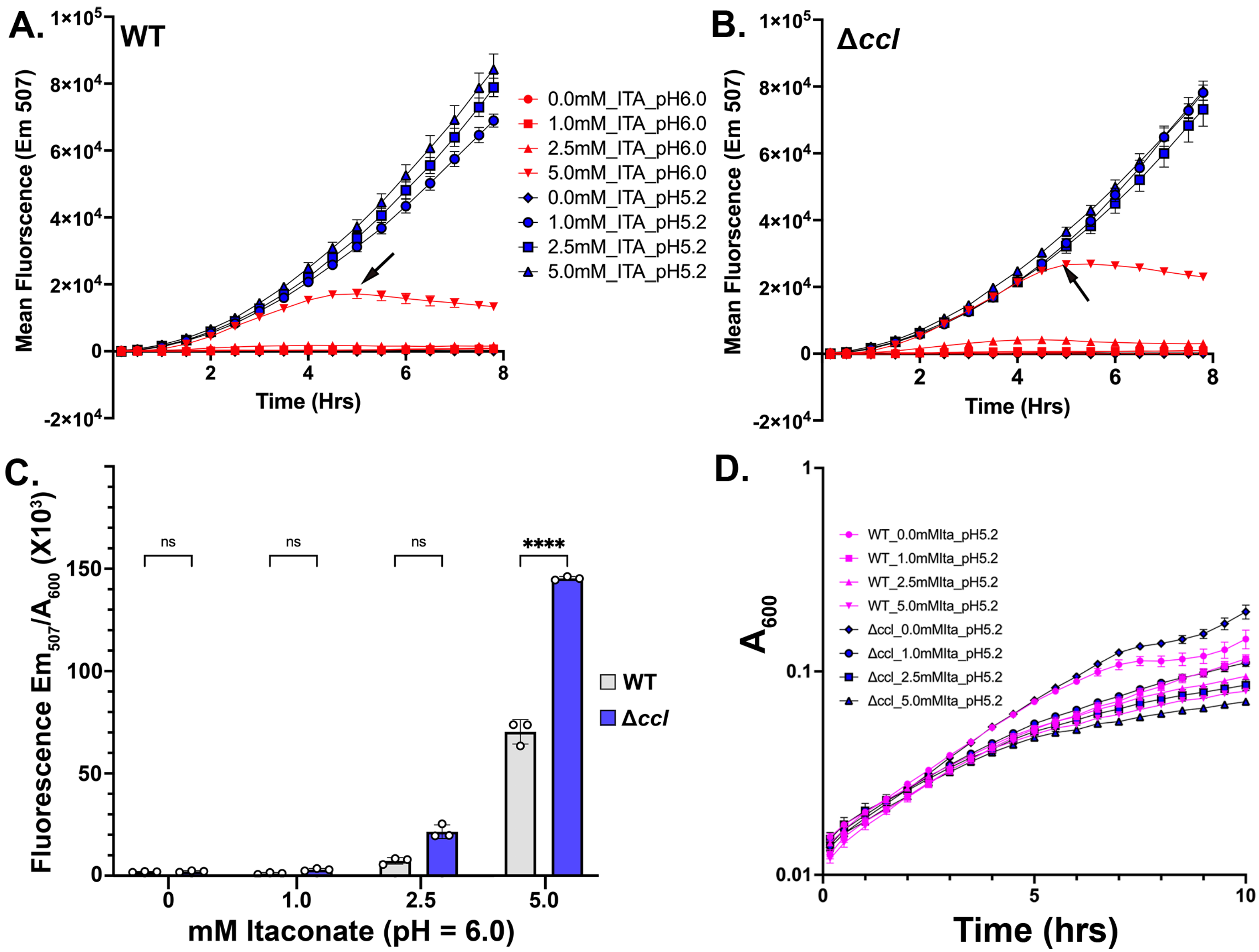

A.
WT
Mean Fluorscence (Em 507)
Time (Hrs)
0.0mM_ITA_pH6.0
1.0mM_ITA_pH6.0
2.5mM_ITA_pH6.0
5.0mM_ITA_pH6.0
0.0mM_ITA_pH5.2
1.0mM_ITA_pH5.2
2.5mM_ITA_pH5.2
5.0mM_ITA_pH5.2
B.
Δccl
Mean Fluorscence (Em 507)
Time (Hrs)
C.
Fluorescence Em507/A600 (X10^3)
ns
ns
ns
****
WT
Δccl
mM Itaconate (pH = 6.0)
D.
A600
WT_0.0mMIta_pH5.2
WT_1.0mMIta_pH5.2
WT_2.5mMIta_pH5.2
WT_5.0mMIta_pH5.2
Δccl_0.0mMIta_pH5.2
Δccl_1.0mMIta_pH5.2
Δccl_2.5mMIta_pH5.2
Δccl_5.0mMIta_pH5.2
Time (hrs)

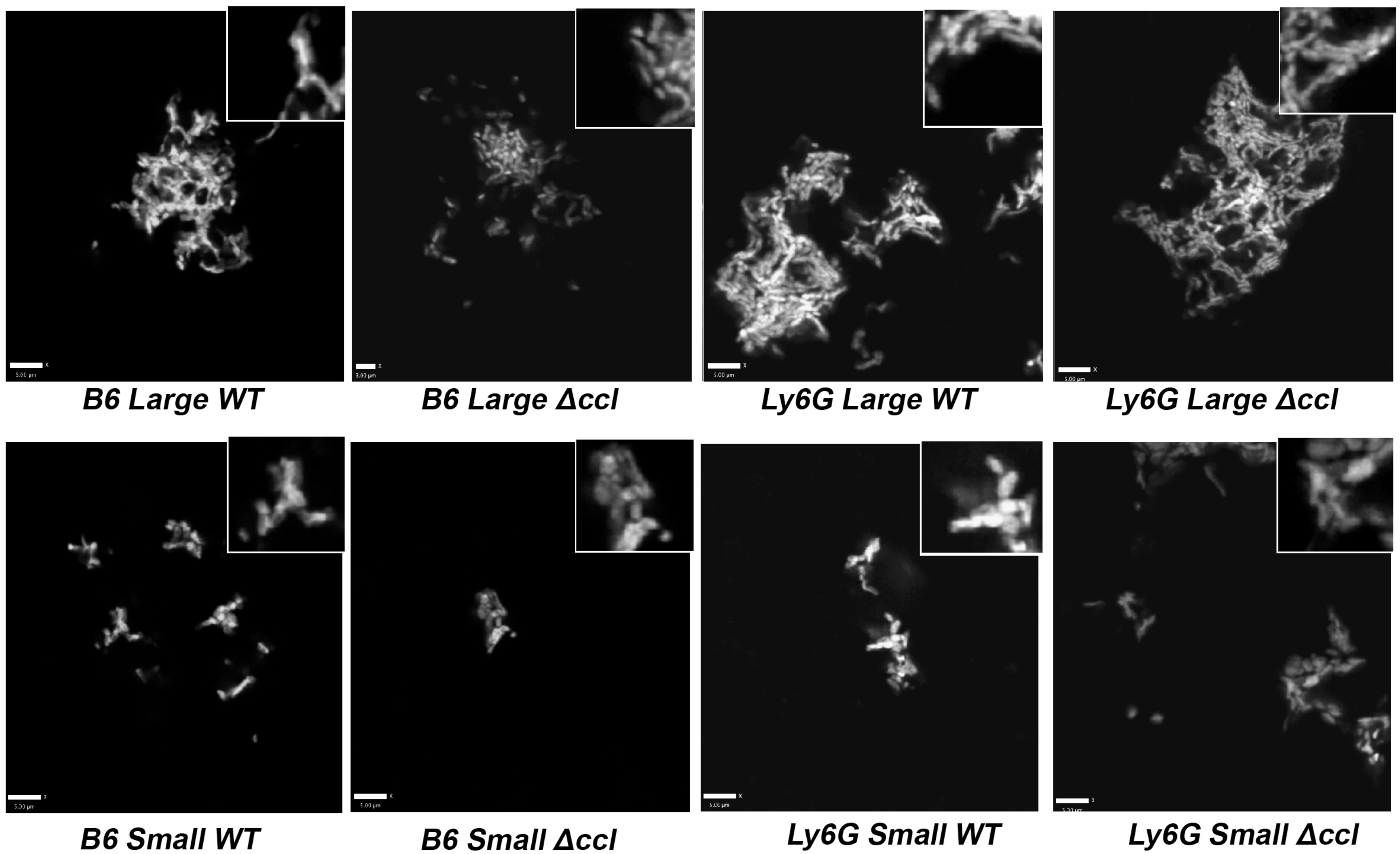
B6 Large WT
B6 Large Δccl
Ly6G Large WT
Ly6G Large Δccl
B6 Small WT
B6 Small Δccl
Ly6G Small WT
Ly6G Small Δccl
