# Supplementary material for: The transcriptional response of Yersinia pseudotuberculosis to macrophage-released metabolites during growth within synthetic microcolonies
Source: mBio. 2026 Sep 21;17(10):e00691-26. doi: 10.1128/mbio.00691-26 (PMC13712848; doi:10.1128/mbio.00691-26)
Supplement: Supplementary file 2 [file mbio.00691-26-s0004.docx]

**Supplemental Figure 1. The presence of RNA*later* results in loss of emission from fluorescent proteins.** Overnight broth cultures of *Yptb gfp+ Phmp::mcherry* were back diluted 1:100 in 2xYT and grown in the presence of 2.5 mM DETA-NONOate for 4 hours prior to applying the agarose/ HyStem^®^-C Hydrogel droplet dispersal protocol (18) in the presence of PBS or RNA*later*, and fluorescence was analyzed after each step in the protocol by flow cytometry. Flow fluorescence profiles are as follows. (1) Pelleted bacterial cells resuspended in 100 μl PBS or RNA*later* (RNA*later* Treatment); (2) RNA*later* Treatment in presence of incubation at 50^o^C for 3 min. (3) RNA*later* Treatment in presence of addition of collagenase, hyaluronidase, and agarase. (4) RNA*later* Treatment in presence of incubation step 37 ^o^C for 45 min. (5.) RNA*later* Treatment in presence of incubation step 60 ^o^C for 3 min.

**Supplemental Figure 2. Transcriptional response to itaconate in the absence of the *ccl* operon.** WT and Δ(*ccl-ich*-*ict*) *Yptb P_ccl_::gfp* strains were grown at 26^o^C in DMEM/HEPES + 10% FBS adjusted to pH = 6.0 (blue symbols) or pH = 5.2 (red symbols) in the presence or absence of various concentrations of itaconate in 96 well plates. Fluorescence of GFP was continuously monitored (ex=485nm, em=507nm; Materials and Methods) and plotted as a function of time. (A). Expression of *P_ccl_* promoter in WT strain background in response to noted itaconate concentrations (0.0, 1.0, 2.5 and 5.0 mM in DMEM/HEPES+10% FBS) and pH conditions as monitored by GFP fluorescence. (B). Expression of *P_ccl_* promoter in Δ(*ccl-ich*-*ict*) strain background in response to noted itaconate concentrations (.0, 2.5 and 5.0 mM in DMEM/HEPES+10% FBS) and pH conditions as monitored by GFP fluorescence. (C). Hyperexpression of the *P_ccl_* promoter in the absence of the itaconate degradation pathway in medium adjusted to pH = 6.0. Data from the 5 hour timepoint from panels A and B (noted by arrows) were plotted to compare the response of the WT and Δ(*ccl-ich*-*ict*) strain to itaconate concentrations. (D). Relative growth inhibition at low pH of *Yptb* strains. WT and Δ(*ccl-ich*-*ict*) *Yptb P_ccl_::gfp* strains were grown at 26^o^C in DMEM/HEPES+10% FBS medium adjusted to pH = 5.2 in the presence or absence of noted concentrations of itaconate in 96 well plates. Growth was monitored by reading absorbance at A_600_ and plotted as a function of time.

**Supplemental Figure 3.** Small colony size is associated with bloated bacterial morphology. Shown are colonies from spleen sections qualitatively scored as small and large then imaged for bacterial morphology. The noted bacterial strains (*Yptb-gfp* WT and Δ(*ccl-ich*-*ict*)) were infected as described (Materials and Methods) into C57Bl/6 mice in the absence or presence of neutrophil depletion with anti-Ly6G. After euthanization, colony sizes were measured (Fig. 8) and GFP fluorescence imaged.
